# Radiation-induction of MMPs limited the NK-mediated anticancer immunity in lung cancer cells

**DOI:** 10.1186/2049-3002-2-S1-P26

**Published:** 2014-05-28

**Authors:** Woong Heo, Young Shin Lee, Cheol Hun Son, Kwang Mo Yang, You Soo Park, Jae Ho Bae

**Affiliations:** 1 Department of Biochemistry, Pusan National University School of Medicine, Yangsan, Republic of Korea; 2 Department of Research Center, Dongnam Institute of Radiological & Medical Sciences, Pusan, Republic of Korea

## 

Radiotherapy has been used to treat cancer and is required to treat many cancer patients. Although radiotherapy effectively inhibits the growth of cancer cells by inducing cell death, it is necessary to investigate its adverse effects such as promotion of cancer metastasis. It has been known that matrix metalloproteinases (MMPs), which play an important role in invasion of cancer cells, were induced by irradiation and are associated with the prognosis of some cancer patients. Recently, this induction of MMPs increased the shedding of natural killer group 2 member D ligands (NKG2DLs) and decreased surface expression of NKG2DLs on cancer cells. As a consequence, the cancer cells escape NK-mediated anti-cancer immunity. Irradiation induced the expression of MMPs. Previously, despite significant induction of NKG2DLs mRNA after irradiation, discordance appeared between mRNA and surface protein levels and the cytotoxic lysis of cancer cells. It was known that NK cells in cancer patients were inhibited by soluble NKG2DLs(sNKG2DLs), and MMPs had an important role in increasing sNKG2DLs. Therefore, it may be necessary to reduce the sNKG2DLs for NK cell-mediated immunotherapy. We inhibited the activity of MMPs using GM6001 or MMP inhibitor III after irradiation. We investigated whether the inhibition of MMPs affects the expression of NKG2DLs after irradiation in cancer cells. The expressions of MMP2 and ADAM10, which might promote invasion of cancer cells and evade NK-mediated anti-cancer immunity through reduction of surface protein levels of NKG2DLs, were increased by irradiation. It was suggested that treatment with MMP inhibitors might minimize the adverse effect of radiotherapy.

